# Supplementary material for: Optimisation of a Novel Bio-Substrate as a Treatment for Atrophic Age-Related Macular Degeneration
Source: Front Bioeng Biotechnol. 2020 May 15;8:456. doi: 10.3389/fbioe.2020.00456 (PMC7243032; doi:10.3389/fbioe.2020.00456)
Supplement: Supplementary file 1 [file Data_Sheet_1.PDF]

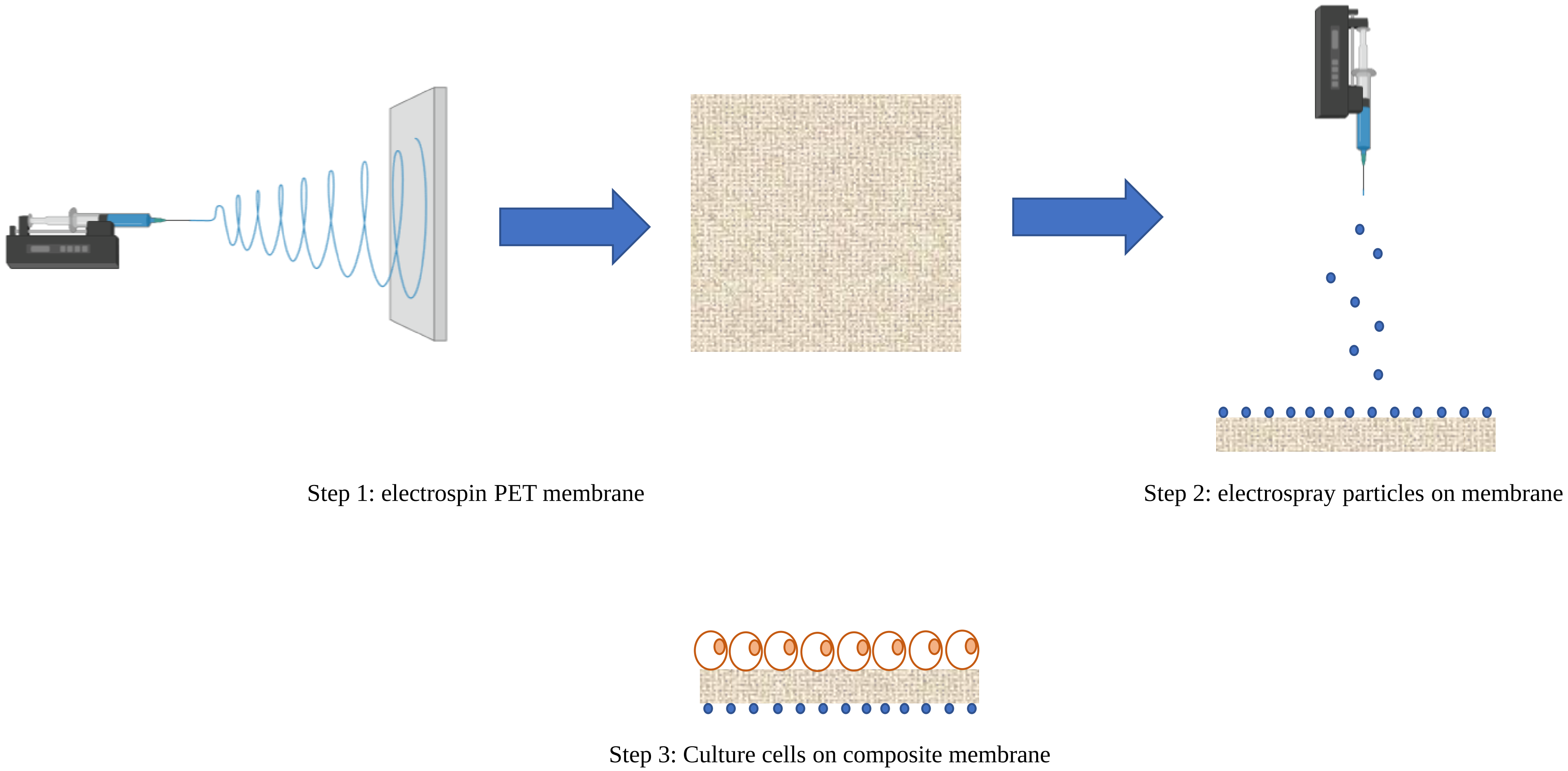

Figure S1: Schematic of the experimental steps leading up to cell culture of composite membrane

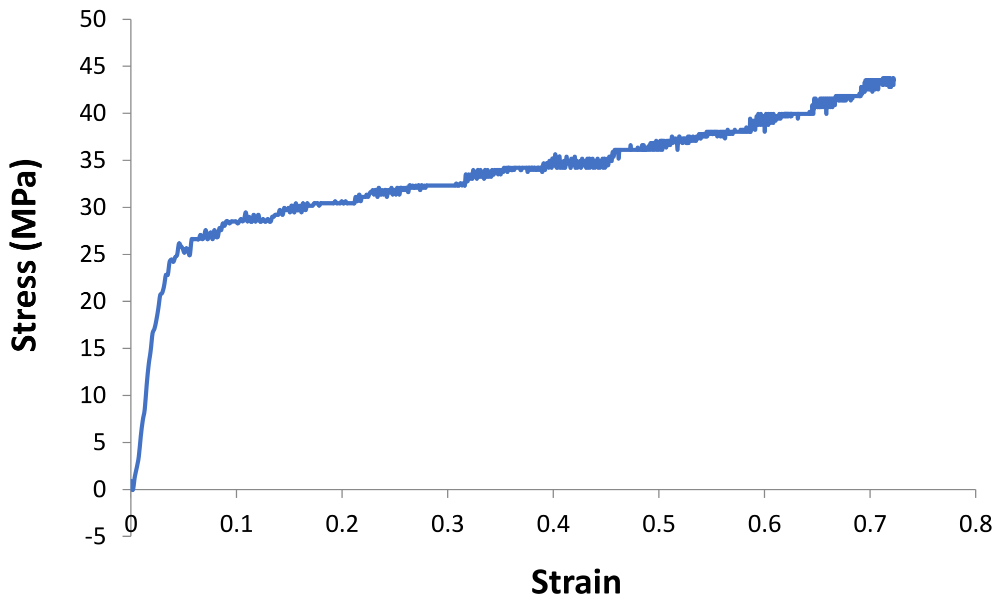

Figure S2. Representative stress vs strain profile of an electrospun membrane sample that did not achieve failure.

Table S1. Denoting the average WCA following treatment, with the media treated PET exhibiting the lowest WCA, 85.8° (+/-20). Data presented: mean (+/- standard error). (n = 6).

**Treatment**	**Average contact angle (°)**
Control	132.6 (+/-11.3)
Media	85.8 (+/-20)
UV	138.1 (+/-6.9)
Ethanol	118.4 (+/-25.4)

Table S2. Effect of degrading nanoparticles on pH of ARPE-19 culture media exhibited little change in pH, with PLGA exhibiting a significant change in week 1 and 2, which resolved thereafter. Data presented: mean (+/- standard error). (n = 6).

**Treatment**	**pH 1 day**	**pH 1 week**	**pH 2 weeks**	**pH 1 month**
Control	7.32 (+/-0.01)	7.43 (+/-0.01)	7.24 (+/-0.001)	7.19 (+/-0.06)
2% PLGA nanoparticles	7.29 (+/-0.02)	7.39* (+/-0.01)	7.16* (+/-0.01)	7.22 (+/-0.03)
1% PGA nanoparticles	7.32 (+/-0.01)	7.441 (+/-0.01)	7.193 (+/-0.03)	7.23 (+/-0.02)

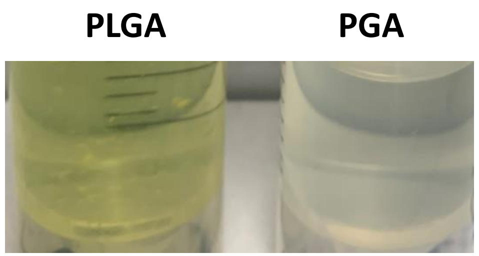

Figure S3. PLGA and PGA nanoparticles encapsulated with FITC and allowed to degrade in 0.1% isopropanol/dH_2O over 28 days. Photographs suggest PLGA released more dye (see figure 14 a-b for comparison).

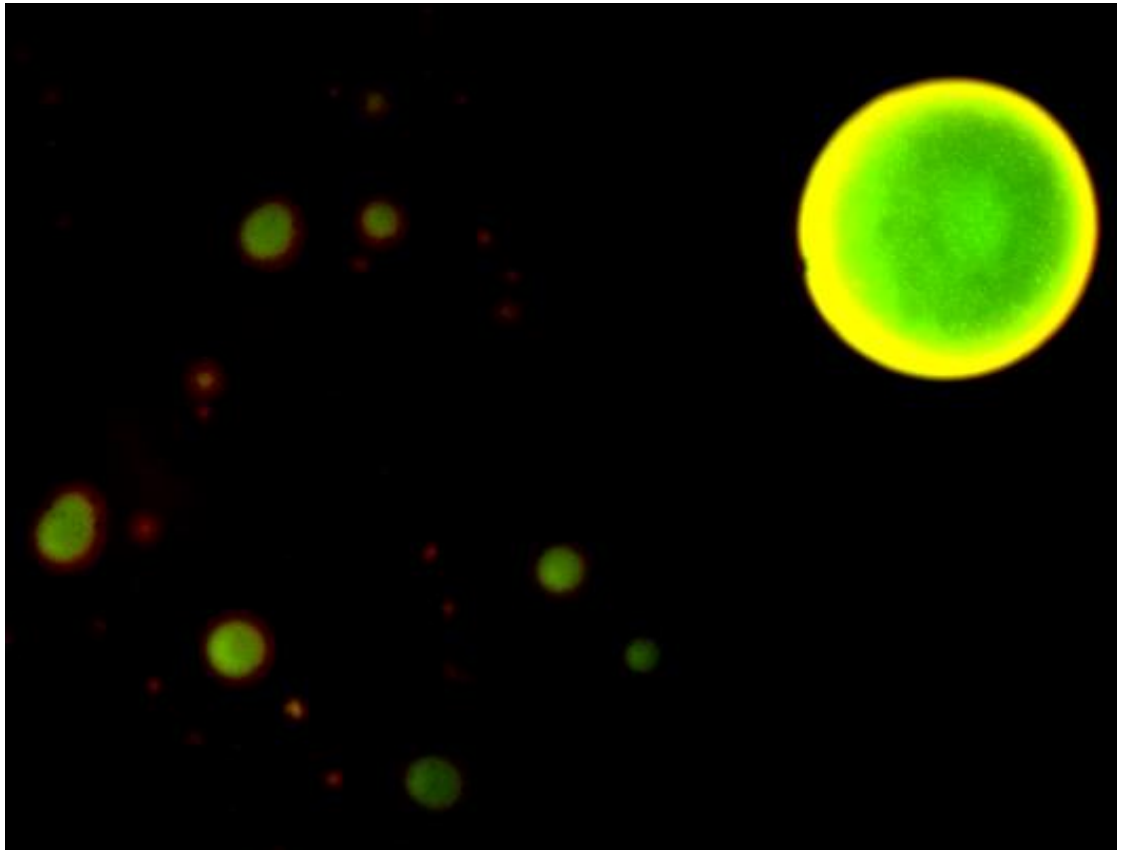

Figure S4. Confocal micrograph of coaxial electrosprayed nanoparticles with an outer shell consisting of 2% PLGA/0.01% Nile red in ethyl acetate encapsulating 0.2% PLGA/1mM FITC in acetone.

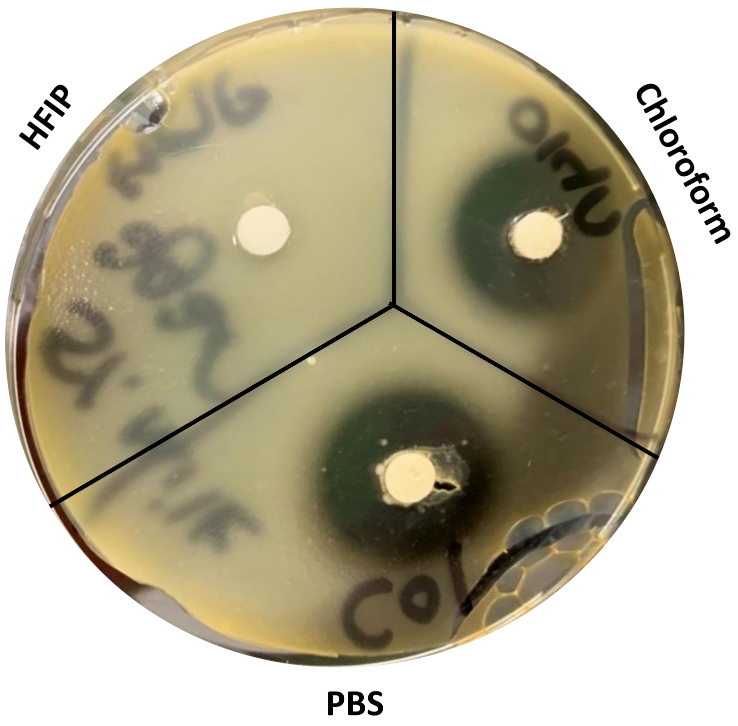

Figure S5. Enzyme activity of collagenase carried out on milk agar assay showed that HFIP denatured the enzyme whereas chloroform did not affect enzyme activity, which was comparable to collagenase dissolved in PBS.
